# Exploring HIV care disparities among foreigners in Taiwan: Insights from a multicenter study (2017-2023)

**DOI:** 10.1016/j.ijregi.2026.100857

**Published:** 2026-02-13

**Authors:** Sung-Hsi Huang, Shu-Ying Chang, Bo-Huang Liou, Chia-Jui Yang, Po-Liang Lu, Luo-Wei Lin, Mei-Hui Lee, Pei-Ying Wu, Chi-Ying Lin, Chung-Yu Shih, Pei-Yu Wang, Mao-Song Tsai, Yuan-Ti Lee, Chien-Yu Cheng, Shu-Hsing Cheng, Chien-Ching Hung, Taiwan HIV Study Group

**Affiliations:** 1 Department of Internal Medicine, National Taiwan University Hospital Hsin-Chu Branch, Hsinchu, Taiwan; 2 Center for International Health, National Taiwan University Hospital Hsin-Chu Branch, Hsinchu, Taiwan; 3 Department of Tropical Medicine and Parasitology, National Taiwan University College of Medicine, Taipei, Taiwan; 4 Department of Infectious Diseases, Taoyuan General Hospital, Taoyuan, Taiwan; 5 Department of Internal Medicine, Hsinchu MacKay Memorial Hospital, Hsinchu City, Taiwan; 6 Department of Medicine, MacKay Medical College, New Taipei, Taiwan; 7 Department of Internal Medicine, Far Eastern Memorial Hospital, New Taipei City, Taiwan; 8 School of Medicine, National Yang Ming Chiao Tung University, Taipei, Taiwan; 9 Department of Internal Medicine, Kaohsiung Medical University Hospital, Kaohsiung, Taiwan; 10 Department of Post-Baccalaureate Medicine, Kaohsiung Medical University, Kaohsiung, Taiwan; 11 School of Medicine, Chung Shan Medical University, Taichung City, Taiwan; 12 Division of Infectious Diseases, Department of Internal Medicine, Chung Shan Medical University Hospital, Taichung City, Taiwan; 13 Division of Infectious Diseases, Department of Internal Medicine, Shuang Ho Hospital, Taipei Medical University, New Taipei City, Taiwan; 14 Center for Infection Control, National Taiwan University Hospital, Taipei, Taiwan; 15 Department of Internal Medicine, National Taiwan University Hospital Yunlin Branch, Yunlin, Taiwan; 16 Center of Infection Control, Far Eastern Memorial Hospital, New Taipei City, Taiwan; 17 Center of Infection Control, National Taiwan University Hospital Hsin-Chu Branch, Hsinchu, Taiwan; 18 School of Medicine, College of Medicine, Fu Jen Catholic University, New Taipei City, Taiwan; 19 Department of Research and Development, Taoyuan General Hospital, Taoyuan, Taiwan; 20 School of Public Health, College of Public Health, Taipei Medical University, Taipei, Taiwan; 21 Department of Internal Medicine, National Taiwan University Hospital and National Taiwan University College of Medicine, Taipei, Taiwan

**Keywords:** HIV, Care continuum, Migrant health, Antiretroviral therapy, Retention in care, Viral suppression

## Abstract

**Objectives:**

The proportion of foreigners among newly diagnosed people with HIV in Taiwan has steadily increased. We evaluated HIV care quality indices among foreigners with HIV (FWH) and explored associated factors.

**Methods:**

FWH who first sought care at 10 Taiwanese hospitals between 2017 and 2023 were included. Information on AIDS at presentation, antiretroviral therapy (ART) prescription, care retention, and viral suppression was collected. Factors associated with these indices were analyzed using logistic regression and Cox proportional hazards models.

**Results:**

We included 144 FWH (median age of 32 years); 74.3% were from Southeast Asia. Overall, 58.3% remained in care at 1 year. Among 94 ART-naïve individuals, 47.9% presented with AIDS and 83.0% initiated ART; however, only 42.6% achieved viral suppression at 1 year. In multivariable analysis, white-collar occupations (vs blue-collar or unemployed) were associated with ART initiation (adjusted odds ratio [aOR], 5.481), while opportunistic infections decreased the odds (aOR, 0.284). ART prescription predicted care retention (adjusted hazard ratio, 6.03), and government-subsidized ART (vs self-funded ART) was linked to higher viral suppression rates (aOR, 3.817).

**Conclusions:**

Substantial gaps in the HIV care cascade were observed among FWH in Taiwan. Strategies to promote earlier diagnosis and improve care retention are urgently needed.

## Highlights

• Late diagnosis is very common in foreigners with newly diagnosed HIV in Taiwan.
• Less than half of foreigners with newly diagnosed HIV remained in care at 1 year.
• Blue-collar work, joblessness, and opportunistic infections reduced therapy uptake.
• Those who started antiretroviral therapy were more likely to stay in care at 1 year.
• Government-funded treatment improved the likelihood of viral suppression.

## Graphical Abstract

**Figure:**
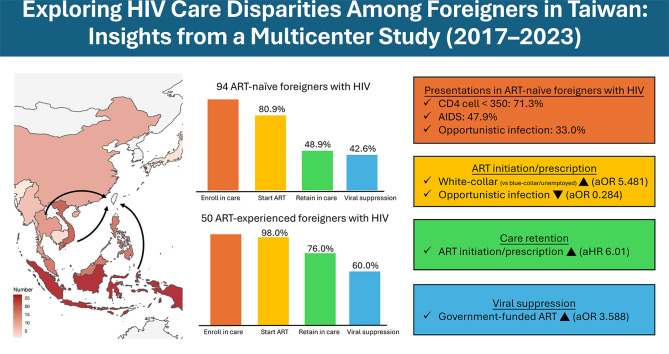

## Introduction

Since the first HIV case was diagnosed in 1984, HIV care delivery and control has significantly improved in Taiwan. HIV testing is widely available through multiple channels, including voluntary counseling and testing and home-based self-testing [1]. People who are Taiwanese nationals and test positive for HIV have free access to HIV care, including state-of-the-art antiretroviral therapy (ART) and cluster of differentiation 4 (CD4) count and plasma HIV RNA monitoring, while those who test negative can employ different preventive measures from HIV acquisition, including HIV pre-exposure prophylaxis (PrEP) [2].

With these collective measures, Taiwan has achieved “92-96-95” of the UNAIDS “95-95-95” targets by the end of 2024 and the annual number of incident HIV cases has declined steadily since 2018 [1,3]. Nevertheless, over the past decade, foreigners have accounted for an increasing proportion of new HIV diagnoses annually in Taiwan, increasing from 1.7% (42 of 2394 cases) in 2016 to 10.8% (121 of 1121 cases) in 2024 [4]. Compared to Taiwanese people with HIV (PWH), foreigners with HIV (FWH) face various additional challenges [5]. In the earlier years, foreign residents were subject to mandatory HIV testing and deportation if found HIV-positive [6], which prevented many from accessing HIV testing or care due to fear of deportation to this date. Although the restrictions on the entry and stay of FWH in Taiwan were lifted in 2015 [7], many barriers to HIV care remain, including a high cost of ART and laboratory testing, fear of job loss or employment insecurity, and the lack of access to health education due to language and social factors [8,9]. These barriers could hinder FWH from HIV testing, care engagement, and achievement of viral suppression, thereby contributing to a sustained HIV viral burden in the community.

As HIV recognizes no social or racial boundaries, the goal of sustained HIV control in Taiwan would not be achieved without attending to this underserved population. In this study, we aimed to evaluate the HIV care delivery among FWH in Taiwan by looking at different indices in the care cascade and to investigate potential factors associated with the key indices of ART initiation, care retention, and viral suppression.

## Methods

### Study design and procedure

In this retrospective study, foreigners with confirmed HIV infection, including migrant workers, immigrants (with or without legal resident status), and students, who were born outside of Taiwan and accessed HIV care services for the first time between 2017 and 2023 at 10 hospitals designated for HIV care in Taiwan were identified through hospital HIV registries. The demographics and the key HIV-related clinical information and laboratory data were collected in a case report form, including the dates of HIV diagnosis, enrollment in HIV care, ART initiation, and last clinical follow-up, ART and its funding source, the initial and follow-up HIV RNA test results, the status of viral hepatitis and syphilis, and any diagnoses of opportunistic infections. Included FWH were observed until mortality, loss to follow-up, transfer out, or the end of study observation period on December 31, 2024, whichever occurred first. The study was approved by the Research Ethics Committees or Institutional Review Boards of the 10 participating hospitals. Informed consent was waived. The study was carried out in accordance with the approved ethical guidelines and regulations.

### Study setting

In Taiwan, HIV infection and AIDS are notifiable diseases and HIV/AIDS care services, including ART, necessary laboratory tests, and management of AIDS-defining conditions, are provided to Taiwanese PWH free-of-charge at more than 90 designated healthcare facilities across Taiwan. However, foreigners are not eligible for this subsidy in the first 2 years of their HIV diagnoses unless they are spouses of Taiwanese nationals, people who acquire HIV during medical procedures or blood transfusion in Taiwan, or specific government-recognized groups, such as Tibetan refugees and descendants of nationalist military personnel from Thailand and Myanmar [10]. Therefore, for most FWH in Taiwan, HIV treatment and follow-up, including ART and plasma HIV RNA testing, are covered by the National Health Insurance (NHI) only after 2 years of their HIV diagnosis and notification to the Taiwan Centers for Disease Control (TCDC) [11].

### Definitions

In this study, we grouped occupations into four categories: (1) “blue-collar” occupations, including labor or service jobs; (2) “white-collar” occupations, including professional, managerial, administrative, or clerical positions; (3) “students or dependents,” referring to individuals who came to Taiwan mainly to study or stayed as dependents of Taiwanese citizens; and (4) “unemployed,” referring to those without a recorded formal occupation. Mandarin proficiency was determined by HIV care providers if relevant HIV and ART counseling could be conducted in Chinese language. Confirmed HIV infection was made by testing positive for HIV nucleic acid amplification test, immunochromagraphic test, or Western blot [12]. In compliance with the definitions by TCDC, acute HIV infection was defined as either having any negative or indeterminate HIV testing within 180 days before confirmed HIV diagnosis, or having negative or indeterminate HIV antibody testing within 180 days before or after confirmed HIV diagnosis. In newly diagnosed FWH, initiating ART within 7 days of enrollment in HIV care was considered rapid ART initiation [13], while late HIV presentation was defined by CD4 cell counts of <350 cells/µl or AIDS-defining illness at the time of presentation. Having regular clinical visits was defined by a maximal interval between clinical follow-ups of <120 days, as prescriptions of no longer than 90 days being the common practice in Taiwan due to NHI regulations. Follow-up interruption was defined by having any intervals between clinical visits of >180 days. HIV viral suppression was defined by a plasma HIV RNA load of <200 copies/ml.

### Statistical analysis

Statistical analysis was performed using the R statistics software (version 4.5.1) through the RStudio interface. Included FWH were divided into two cohorts according to their ART status at the time of HIV care enrollment: the ART-naïve cohort and the ART-experienced cohort. The demographic, clinical, and laboratory characteristics of the included FWH were summarized using medians and interquartile ranges (IQRs) for continuous variables, while categorical variables were described using frequencies and percentages.

The rate of ART initiation (or re-initiation/refill in the ART-experienced cohort), and rates of retention in care, viral load testing, and viral suppression at 1 year were determined. In ART-naïve cohort, duration from care enrollment to ART initiation was calculated. Univariable analyses were performed to screen for factors associated with ART initiation, HIV care retention, and viral suppression. Non-categorical variables were compared using Kruskal-Wallis test and categorical variables were compared using Pearson’s chi-squared test and Fisher’s exact test when applicable.

Multivariable logistic regression was conducted to identify factors associated with ART prescription and with viral suppression in FWH who started ART. Candidate predictors included age, sex, year of enrollment in care, occupation, and Mandarin proficiency, plus any variables with *P* <0.1 in univariable screening. We used Firth bias-reduced logistic regression to cope with small sample size and complete or quasi-separation in some of the variables and results were reported as adjusted odds ratios (aORs) with 95% confidence intervals (CIs).

A Kaplan-Meier plot was depicted to demonstrate time to care discontinuation and group differences were compared using the log-rank test. Variables including age, sex, year of enrollment in care, occupation, Mandarin proficiency, and variables with *p*-value <0.1 in univariable analysis, were included in Cox proportional hazards model to investigate independent factors associated with HIV care retention at 1 year. *P*-values of <0.05 were deemed statistically significant throughout the analyses.

## Results

The study included a total of 144 FWH, among whom 94 were ART-naïve and 50 were ART-experienced. The median age was 31.9 years, 25% were female, including two individuals who were diagnosed with HIV during pregnancy, and most (74.3%) were from Southeast Asia (Table 1 and Supplementary Figure 1). Almost 60% were migrant workers and 25% came to Taiwan as students or dependents (of his/her family). Less than half were able to communicate in Mandarin.

**Table 1:** Baseline characteristics, clinical presentations, and laboratory findings of 144 included foreigners living with HIV.

	Total	ART-naïve	ART-experienced
	N = 144	N = 94	N = 50
Age, year, median (IQR)	31.9 (27.2, 40.0)	32.1 (27.0, 39.4)	31.8 (27.5, 42.3)
<25	22 (15.3)	17 (18.1)	5 (10.0)
25-34	66 (45.8)	40 (42.6)	26 (52.0)
35-44	32 (22.2)	24 (25.5)	8 (16.0)
≥45	24 (16.7)	13 (13.8)	11 (22.0)
Male sex assigned at birth, n (%)	108 (75.0)	68 (72.3)	40 (80.0)
Risk groups for HIV transmission, n (%)			
Men who have sex with men	80 (55.6)	55 (58.5)	25 (50.0)
Heterosexual	56 (38.9)	33 (35.1)	23 (46.0)
Others^a^	2 (1.4)	1 (1.1)	1 (2.0)
Unknown	6 (4.2)	5 (5.3)	1 (2.0)
Occupation, n (%)			
Blue-collar	53 (36.8)	38 (40.4)	15 (30.0)
White-collar	32 (22.2)	19 (20.2)	13 (26.0)
Student or family	37 (25.7)	24 (25.5)	13 (26.0)
Unemployed	22 (15.3)	13 (13.8)	9 (18.0)
Having a Taiwanese partner, n (%)	49 (34.0)	30 (31.9)	19 (38.0)
Continent of origin, n (%)			
Asia	126 (87.5)	85 (90.4)	41 (82.0)
Southeast Asia	107 (74.3)	72 (76.6)	35 (70.0)
Africa	9 (6.2)	4 (4.3)	5 (10.0)
America	6 (4.2)	3 (3.2)	3 (6.0)
Europe	3 (2.1)	2 (2.1)	1 (2.0)
Country of origin by income level, n (%)			
Low-middle income	58 (40.3)	40 (42.6)	18 (36.0)
High-middle income	74 (51.4)	47 (50.0)	27 (54.0)
High income	12 (8.3)	7 (7.4)	5 (10.0)
Year of enrollment in care, n (%)			
2017-2020	64 (45.5)	49 (52.1)	15 (30.0)
2021-2023	80 (55.6)	45 (47.9)	35 (70.0)
Mandarin proficiency, n (%)	68 (47.2)	41 (43.6)	27 (54.0)
Clinical presentations			
Acute retroviral syndrome, n (%)	–	6 (6.4)	–
AIDS, n (%)	48 (33.3)	45 (47.9)	3 (6.0)
Any opportunistic infections, n (%)	32 (22.2)	31 (33.0)	1 (2.0)
Pneumocystosis, n (%)	13 (9.0)	12 (12.8)	1 (2.0)
Tuberculosis, n (%)	11 (7.6)	10 (10.2)	1 (2.0)
Cryptococcosis, n (%)	3 (2.1)	3 (3.2)	0
Any sexually transmitted infections, n (%)	26 (18.1)	20 (21.3)	6 (12.0)
Laboratory findings at the time of enrollment in HIV care			
Rapid plasma regain ≥1:4, n (%) [N = 143]	13 (9.1)	8 (8.6)	5 (10.0)
Hepatitis B surface antigen positivity, n (%) [N = 142]	3 (2.1)	3 (3.2)	0 (0.0)
Anti-hepatitis C antibody positivity, n (%) [N = 143]	6 (4.2)	5 (5.3)	1 (2.0)
Plasma HIV RNA, median (IQR), log_10_ copies/ml [N = 135]	4.5 [1.3, 5.2]	5.1 [4.5, 5.8]	1.3 [1.3, 1.7]
HIV RNA load >100,000 copies/ml, n (%)	49 (36.3)	48 (55.8)	1 (2.0)
HIV RNA load <200 copies/ml, n (%)	42 (31.1)	3 (3.5)	39 (79.6)
CD4 lymphocyte counts, median (IQR), cells/μl [N = 134]	347 [130, 585]	232 [48, 373]	627 [417, 794]
CD4 <350 cells/μl, n (%)	68 (50.7)	62 (71.3)	6 (12.8)
CD4 <200 cells/μl, n (%)	45 (33.6)	42 (48.3)	3 (6.4)

a Including people who inject drug and transfusion. ART, antiretroviral therapy; CD, clusters of differentiations; IQR, interquartile range.

### Clinical presentations

At the time of HIV care engagement, 6.4% ART-naïve FWH had acute retroviral syndrome and 71.3% presented with late HIV diagnosis, 47.9% with AIDS, and a third with opportunistic infections (Table 1). Their median CD4 lymphocyte count was 232 cells/µl and plasma HIV RNA load was 5.1 log_10_ copies/ml. Opportunistic infections such as *Pneumocystis jirovecii* pneumonia, tuberculosis, and cryptococcosis occurred in 12.8%, 10.2%, and 3.3%, respectively. Five (5.3%) FWH in the ART-naïve group died after a median of 28 days of care (range 3 to 66 days).

In ART-experienced cohort, 39 of 49 (79.6%) had achieved viral suppression at the time of care enrollment. The plasma HIV RNA loads ranged from 2460 to 360,316 copies/ml among 10 individuals with documented viremia at the time of enrollment in care, three had CD4 counts of <200 cells/µl, and one presented with pneumocystosis and tuberculosis.

Diagnoses of any sexually transmitted infections at the time of presentation, including syphilis, viral hepatitis, genital herpes, and/or condyloma, were found in 18.1% of all included FWH. The seroprevalences of syphilis, hepatitis B virus, and hepatitis C virus were 9.1%, 2.1%, and 4.2%, respectively.

### HIV care cascade

**Figure 1:**
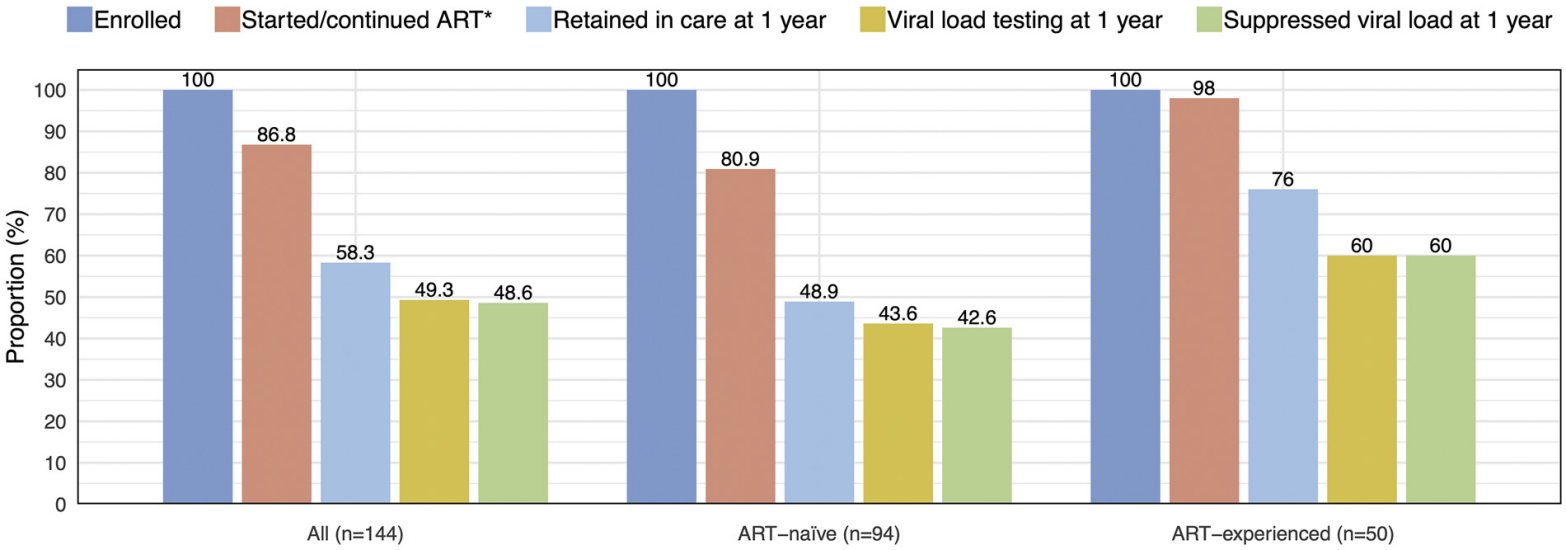
HIV care cascade among 144 foreigners living with HIV in Taiwan, 2017-2023 (Note: asterisk in the figure indicated proportion of individuals starting ART in the ART-naïve cohort and proportion re-starting or continuing ART in the ART-experienced cohort).

Figure 1 illustrates the HIV care cascade of all included FWH, and in ART-naïve and ART-experienced cohorts. Overall, the largest gaps in the care cascade occurred in care retention, with merely 48.9% and 76.0% remained in care at 1 year in ART-naïve and ART-experienced cohorts, respectively. Of those who retained in care at 1 year, 15.5% did not have plasma HIV RNA determined at 1 year, leading to another drop in the cascade. Among ART-naïve FWH, the second and third 95 were 80.9% and 52.7%, respectively.

ART, antiretroviral therapy.

**Figure 2:**
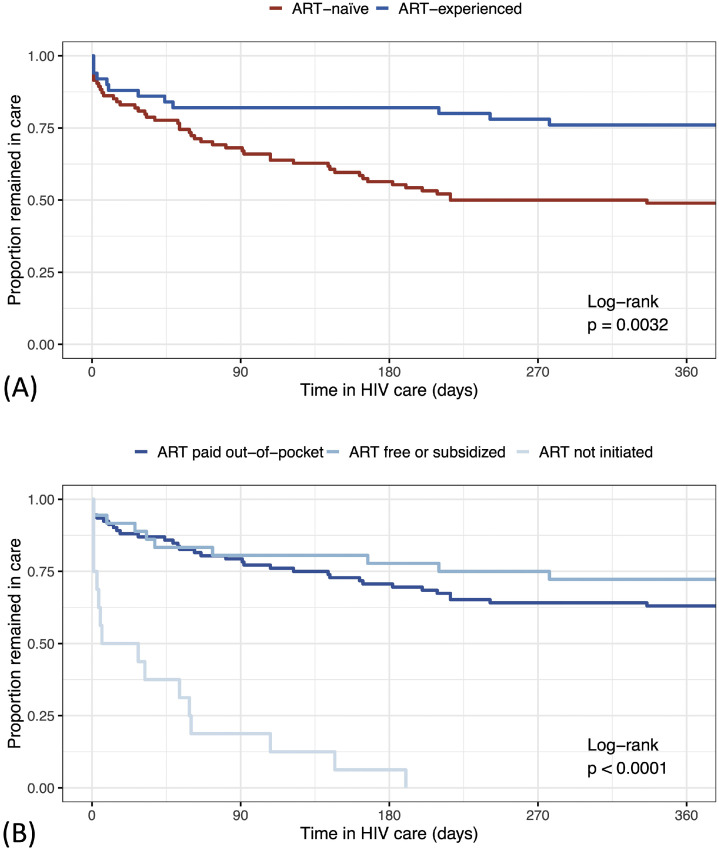
Retention in HIV care during the first year among foreigners with HIV in Taiwan in Kaplan-Meier plots with grouping according to (a) ART status and (b) ART prescription and funding source. ART, antiretroviral therapy.

Figure 2a illustrates the retention in HIV care among ART-naïve and ART-experienced FWH. Among 60 FWH who were disengaged from HIV care within a year, the most common identifiable reasons were returning to the home country (48.3%), transfer to another healthcare facility (10.0%) and mortality (8.3%), while 33.3% were lost to follow-up.

### ART prescription, its associated factors, and HIV RNA load monitoring

Overall, 125 (86.8%) of 144 FWH started, re-started, or continued ART in Taiwan. Multivariable analysis showed higher odds of ART prescription for those enrolled in care during 2021-2023 vs 2017-2020 (aOR 5.600; 95% CI, 1.775-21.328) and for those with white-collar occupations vs those with blue-collar occupations or unemployed (aOR 5.481; 95% CI, 1.049-57.284). In contrast, presentation with an opportunistic infection was associated with lower odds of ART prescription (aOR 0.284; 95% CI, 0.085-0.921) (Table 2). Among the 78 ART initiators in the ART-naïve cohort, median time from enrollment to ART initiation was 4 days (IQR, 0-10) and 58.4% started ART within 7 days.

**Table 2:** Factors associated with prescription of antiretroviral therapy in foreigners living with HIV in Taiwan in univariable and multivariable analysis.

	ART not initiated	ART initiated	Univariable analysis	Multivariable analysis	
	N = 19	N = 125	*P*-value	Adjusted odds ratio (95% confidence interval)	*P*-value
Age, years, median (IQR)	31.4 (25.6, 39.4)	32.6 (27.2, 40.7)	0.593	1.010 (0.954-1.083)	0.758
Male sex assigned at birth	15 (78.9)	93 (74.4)	0.887	0.677 (0.133-1.083)	0.628
Enrolled in care in 2021-2023 (vs 2017-2020)	5 (26.3)	75 (60.0)	0.012	5.600 (1.775-21.328)	0.003
Risk factors			0.085		
Men who have sex with men	10 (52.6)	70 (56.0)		Reference	–
Heterosexual	6 (31.6)	50 (40.0)		1.386 (0.333-6.424)	0.657
Others^a^	0 (0.0)	2 (1.6)		0.755 (0.027-126.531)	0.878
Unknown	3 (15.8)	3 (2.4)		0.232 (0.036-1.628)	0.136
Occupation			0.035		
Blue-collar or unemployed	15 (78.9)	60 (48.0)		Reference	–
White-collar	1 (5.3)	31 (24.8)		5.481 (1.049-57.284)	0.043
Student or dependent	3 (15.8)	34 (27.2)		2.478 (0.668-11.465)	0.181
Mandarin proficiency	6 (31.6)	62 (49.6)	0.223	2.315 (0.759-7.723)	0.142
Presenting with any opportunistic infections	9 (47.4)	23 (18.4)	0.014	0.284 (0.085-0.921)	0.036

a Including people who inject drug and transfusion. ART, antiretroviral therapy; IQR, interquartile range.

The prescribed ART included integrase strand transferase inhibitor-based (63%), non-nucleoside reverse transcriptase inhibitor-based (35%) and others (2%). Most of these medications were paid out-of-pocket (72%), while other ART initiators received reimbursement from Taiwanese government (16.8%), donation (6.4%), or ART shipped from their home countries (4.8%). Generic formulations accounted for 20.8% of ART used by the FWH in this study.

The 84 FWH who remained in care at 1 year underwent a median of 2 HIV RNA load testing (IQR, 1-3) within the 12-month period. Among these individuals, only 48.8% had regular clinical visits (at least once every 120 days) and 27.4% had follow-up interruptions during a median observation period of 917 days (IQR, 603.5-1297.2 days).

### Factors associated with care retention and HIV viral suppression

Several factors were found to be associated with retention in care at 1 year in univariable analysis, including year of enrollment, occupation, Southeast Asian origin, AIDS at presentation, and ART prescriptions (Supplementary Table 1). In a Cox proportional hazards model, ART non-initiation was the strongest and the only independent associated factor with time to care discontinuation (adjusted hazard ratio 6.01 [95% CI 3.06-11.83] and *P*-value <0.001) (Supplementary Figure 2). A Kaplan-Meier plot demonstrated that those who started ART, either through various funding parties or through their own expense, were more likely to remain in care (*p*-value <0.001) (Figure 2b).

Among FWH who started ART, the multivariable analysis showed that those who received ART subsidized by the Taiwanese government had a higher chance to achieve virological suppression at 1 year compared to those who received ART out of their own expense (aOR 3.588, 95% CI 1.145-13.468) (Table 3).

**Table 3:** Factors associated with viral suppression at 1 year in foreigners living with HIV who were on antiretroviral therapy in univariable and multivariable analysis.

	FWH without virological suppression	FWH with virological suppression	Univariable analysis	Multivariable analysis	
	N = 56	N = 69	*P*-value	Adjusted odds ratio	*P*-value
Age, years, median (IQR)	30.0 (26.6, 36.0)	34.5 (28.3, 43.3)	0.011	1.032 (0.995-1.074)	0.094
Male sex assigned at birth	45 (80.4)	48 (69.6)	0.242	0.879 (0.341-2.244)	0.787
Enrolled in care in 2021-2023 (vs 2017-2020)	30 (53.6)	45 (65.2)	0.255	1.812 (0.810-4.161)	0.149
Occupation			0.490		
Blue-collar or unemployed	26 (46.4)	34 (49.3)		Reference	–
White-collar	12 (21.4)	19 (27.5)		1.262 (0.489-3.311)	0.631
Student or dependent	18 (32.1)	16 (23.2)		0.834 (0.331-2.109)	0.699
Mandarin proficiency	25 (44.6)	37 (53.6)	0.413	1.332 (0.596-3.016)	0.485
ART funding source			0.002		
ART paid out-of-pocket	44 (78.6)	46 (66.7)		Reference	**–**
ART subsidized by Taiwanese government	4 (7.1)	17 (24.6)		3.588 (1.145-13.468)	0.028
ART donated	7 (12.5)	1 (1.4)		0.229 (0.023-1.132)	0.073
ART from home country	1 (1.8)	5 (7.2)		1.881 (0.300-20.181)	0.515

ART, antiretroviral therapy; FWH, foreigners living with HIV; IQR, interquartile range.

## Discussion

In this first study focusing on HIV care cascade in foreigners in Taiwan, we identified significant gaps in ART initiation, care retention, and viral load monitoring in this population, which led to poor rates of viral suppression. Blue-collar occupations, unemployment, and opportunistic infections were associated with failure to initiate ART, which was the most important risk factor for HIV care discontinuation. Government subsidies for ART and viral load monitoring was associated with improved HIV viral suppression.

Suboptimal HIV care indices among foreigners were not unique in Taiwan as they had been reported in several high-income countries. For example, in Australia and Italy, migrants with HIV were less likely to start ART and more likely to experience virologic failure [14,15]. Our studies highlighted many discrepancies in HIV care indices between foreigners in Taiwan and Taiwanese PWH. At the end of 2023, more than 95% of diagnosed Taiwanese PWH started ART, and 95% of whom achieved viral suppression [1]. In comparison, among FWH in Taiwan, these numbers declined sharply to 80.9% and 52.7%, respectively. After endorsement of rapid ART by the World Health Organization guidelines in 2017, 80% of newly diagnosed Taiwanese PWH started ART within 7 days of their diagnosis [13], while only 58.4% of FWH who initiated ART in this study did so. Our study also showed less frequent viral load monitoring (median two viral load tests among FWH vs four viral load tests commonly prescribed in ART-naïve PWH in Taiwan in the first 2 years), inconsistent clinical follow-up, and common care interruptions among FWH. All of these factors contributed to suboptimal viral control. With the growing number of FWH in Taiwan and the ongoing risk of viral transmission, these care discrepancies can no longer be overlooked, and actions are needed to improve HIV care in this vulnerable population.

Financial barriers remain a major obstacle for FWH in accessing essential HIV services. Many migrant workers in Taiwan face difficulties affording ART and laboratory testing, as these expenses can easily exceed their monthly wages. In our analysis, FWH’s occupation emerged as a key factor associated with ART initiation, which is the first step to viral suppression. Studies showed that low-wage migrant workers tend to encounter greater barriers to healthcare services [16,17], possess limited knowledge in self-care [18], have reduced access to health information [18], and face heightened job insecurity [19]. Consequently, ART and HIV testing are likely to be less affordable and accessible to the FWH with blue-collar occupations or those who are unemployed.

Conversely, our findings indicated that provision of ART, regardless of the funding source, improved retention in care. Moreover, government coverage of ART and viral load monitoring substantially increased the likelihood of successful viral suppression, likely by minimized financial and logistical barriers. To address these challenges, the TCDC launched the “Support Program for Foreign HIV Patients Receiving Medications Within 2 Years in Taiwan” in August 2024 [11]. Sponsored by a pharmaceutical company, this program provides free ART in the first 2 years following HIV diagnosis, along with regular monitoring of HIV viral load and CD4 count. Analysis of the data from this initiative will inform whether such a strategy can enhance care retention and viral control among this vulnerable population.

A qualitative study in Taiwan identified language and information barriers as major hurdles for migrants seeking healthcare, including HIV testing [5]. It also highlighted that the complexity of Mandarin, especially when it comes to medical terminology, and the shortages of trained medical interpreters often impede effective communication between foreigners and healthcare providers. In our study, language proficiency was not significantly correlated with care indices, although the limited sample size prevent firm conclusions. Enhancing HIV-related health literacy remains a central objective in improving care among foreigners in Taiwan. To this end, multilingual online HIV-related health information was disseminated through the TCDC website and several Taiwanese not-for-profit organizations [10,20]. In 2025, a government-sponsored program to train multilingual interpreters for HIV counseling was also begun, aiming to address the persistent language and information barriers in FWH.

In addition to financial and structural barriers, psychosocial support, including mental health care, patient education, and counseling, is critical for engagement and retention in HIV care among vulnerable foreign populations. HIV care in Taiwan is delivered through a nationally regulated, hospital-based system with standardized training and guidelines [1,21]. However, the participating hospitals in this study adopted different models of psychosocial support for foreigners with HIV, including case management, social work, and non-governmental organization collaboration [1]. Due to the retrospective design of this study, such psychosocial and institutional-level interventions were not systematically documented or standardized across centers, and therefore, could not be included in the quantitative analyses. Heterogeneity in the availability and intensity of these supportive services may therefore have influenced retention in care and represents an important unmeasured factor.

HIV diagnosis and care engagement are often delayed among foreigners, probably due to lack of awareness of HIV risk, inadequate information and limited access to screening services, and the fear of stigma and deportation. As a result, late HIV presentations were common in FWH, leading to devastating outcomes and increased financial burden [22,23]. Successful management of such cases typically requires not only effective medical treatment but also substantial social and familial support—resources that many low-wage migrant workers in Taiwan unfortunately lack. A third of the ART-naïve FWH in this study experienced opportunistic infections, many of them required hospitalization, and five died. As illustrated in several case reports [24], the complexity of caring for such patients could be overwhelming, involving challenges such as establishing a diagnosis with limited clinical history, communicating critical and urgent information with different stakeholders while addressing ethical concerns about privacy, and balancing the risks and benefits of repatriation. Compared with Taiwanese PWH [25], the rate of late presentation was higher among FWH, at 71.3%. Also, in the United States and Spain, foreign-born people and immigrants were more likely to have late HIV diagnosis [26,27]. Improving awareness among foreigners at risk and ensuring convenient and friendly access to HIV testing could potentially enhance earlier HIV diagnosis and overall outcome [28].

Our study has several limitations. First, the sample size was small, and the cohort was derived from 10 participating hospitals, representing approximately 16% of foreigners newly diagnosed with HIV in Taiwan during the study period. Although these hospitals were geographically distributed across northern, central, and southern Taiwan and the demographic profile of included foreigners was broadly consistent with national surveillance data, our findings may not be generalizable to all FWH in Taiwan. In addition, because FWH were identified through hospital registries, individuals who faced challenges in accessing testing, counseling, and formal HIV care, including some undocumented migrants or pregnant foreigners, may be underrepresented. Finally, barriers for foreigners to access HIV care are likely multifactorial. Cultural differences [9], psychiatric conditions and support, fear of stigmatization [29], institutional differences in resources and patient volume could not be fully examined in this retrospective study. Larger, prospectively designed studies integrating clinical, psychosocial, and institutional-level data are needed to address these gaps.

By the end of 2024, 948,066 foreign residents were living in Taiwan [30], accounting for approximately 4% of the total population. A considerable proportion of FWH in this cohort presented with concomitant sexually transmitted infections, which underscores the potential for ongoing sexual transmission of HIV in the wider community. In contrast, the small yet notable proportion of FWH were diagnosed during the acute phase of the infection, suggesting that some acquired HIV in Taiwan. Strengthening HIV screening and care among foreigners living with or at risk for HIV will not only advance health equity, but also contribute to broader HIV prevention effort in this country. Encouragingly, improvements in ART prescription with time were observed in this cohort, reflecting growing awareness and investment of the public and private sectors, and an enhanced capacity to provide care to this underserved population. With continued implementation of the initiatives described previously, sustained progress in the care of FWH in Taiwan can be anticipated.

## Conclusion

Substantial gaps in the HIV care cascade persist among FWH in Taiwan. Continued efforts to strengthen HIV education, expand screening, and minimize financial and informational barriers are essential to ensure earlier diagnosis, improved care retention, and greater viral suppression among foreigners in Taiwan.

## Declaration of competing interest

C.-C. H. has received research support from Gilead Sciences, speaker honoraria from Gilead Sciences, and served on advisory boards for Gilead Sciences. Other authors have no competing interest to disclose.

## Appendix Supplementary materials

Image, application 1
