# Supplementary material for: Exploring HIV care disparities among foreigners in Taiwan: Insights from a multicenter study (2017-2023)
Source: IJID Reg. 2026 Feb 13;18:100857. doi: 10.1016/j.ijregi.2026.100857 (PMC12969629; doi:10.1016/j.ijregi.2026.100857)
Supplement: Supplementary file 1 [file mmc1.docx]

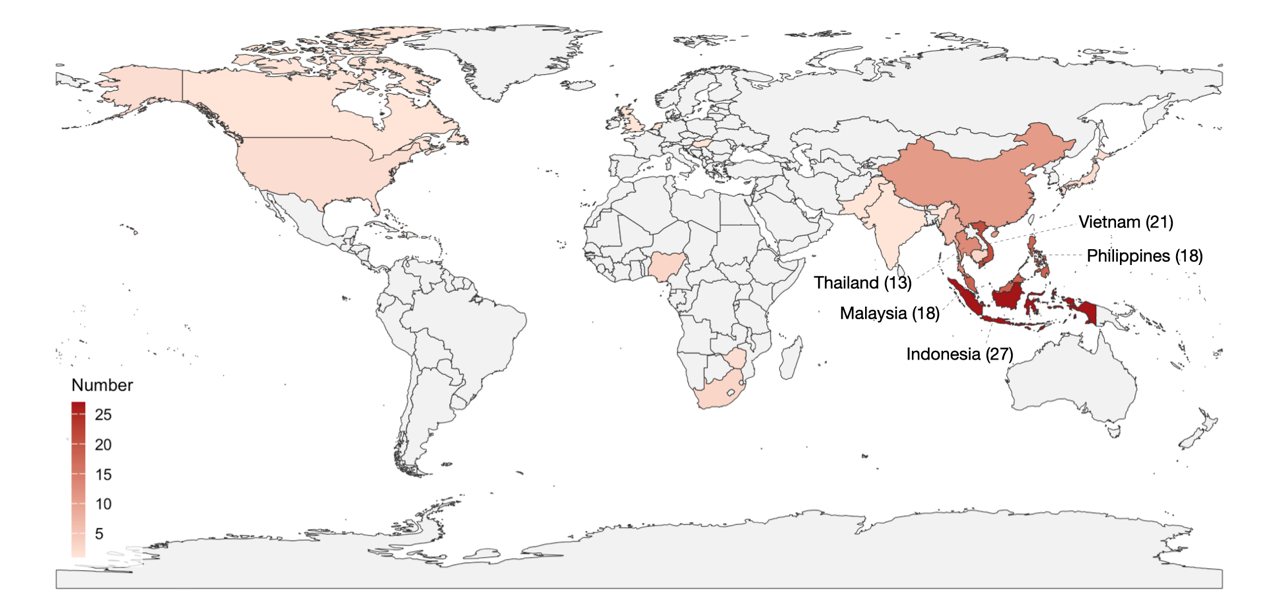
**Supplementary Figure 1**. World map indicating the origin countries of included foreigners living with HIV in Taiwan concentrated in Southeastern Asia.


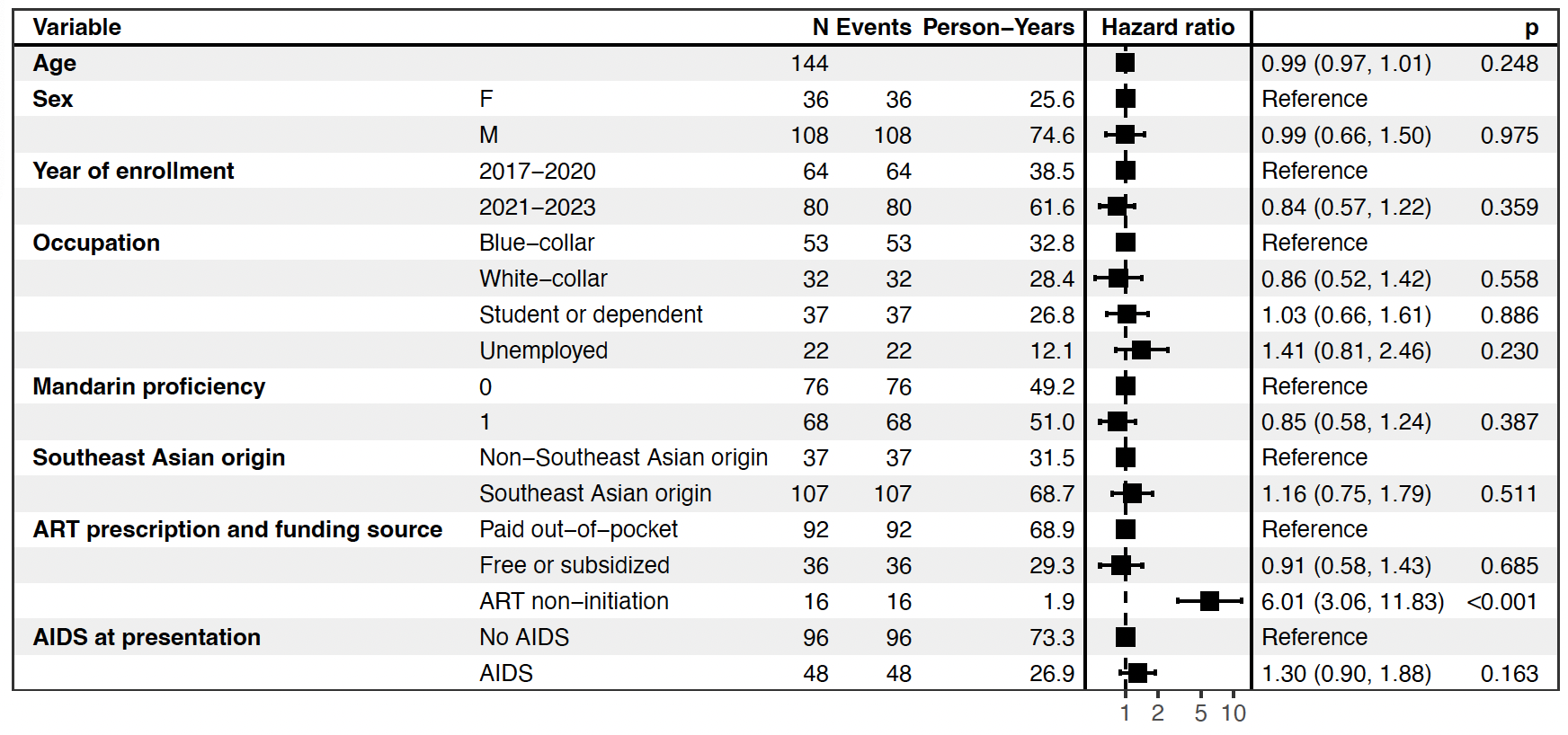
**Supplementary Figure 2**. Factors associated with time to HIV care discontinuation in a Cox proportional hazards model

**Supplementary Table 1.** Factors associated with retention in care at 1 year in foreigners living with HIV in Taiwan in univariable and multivariable analysis

|  | Care discontinuation | Care retention | Univariable analysis |
| --- | --- | --- | --- |
|  | N = 60 | N = 84 | *p*-value |
| Age, years, median (IQR) | 31.4 [26.8, 38.6] | 33.1 [27.6, 42.7] | 0.139 |
| Male sex assigned at birth | 46 (76.7) | 62 (73.8) | 0.845 |
| Year of enrollment |  |  | **0.008** |
| 2017-2020 | 35 (58.3) | 29 (34.5) |  |
| 2021-2023 | 25 (41.7) | 55 (65.5) |  |
| Risk factors |  |  | 0.129 |
| Men who have sex with men | 34 (56.7) | 46 (54.8) |  |
| Heterosexual | 20 (33.3) | 36 (42.9) |  |
| Others^1^ | 1 (1.7) | 1 (1.2) |  |
| Unknown | 5 (8.3) | 1 (1.2) |  |
| Occupation |  |  | **0.035** |
| Blue collar | 25 (41.7) | 28 (33.3) |  |
| White collar | 7 (11.7) | 25 (29.8) |  |
| Student or dependent | 15 (25.0) | 22 (26.2) |  |
| Unemployed | 13 (21.7) | 9 (10.7) |  |
| Having a Taiwanese partner | 17 (28.3) | 32 (38.1) | 0.298 |
| Mandarin proficiency | 24 (40.0) | 44 (52.4) | 0.194 |
| From Southeast Asia countries^2^ | 51 (85.0) | 56 (66.7) | **0.022** |
| Presenting with AIDS | 28 (46.7) | 20 (23.8) | **0.007** |
| ART prescription and fundings source |  |  | **<0.001** |
| ART paid out-of-pocket | 34 (56.7) | 58 (69.0) |  |
| ART free or subsidized | 10 (16.7) | 26 (31.0) |  |
| ART not initiated | 16 (26.7) | 0 (0.0) |  |

^1^ Including people who inject drug and transfusion.

^2^ Including Indonesia, Malaysia, Thailand, Vietnam, Philippines, Myanmar, and Cambodia.

**Abbreviations**: ART, antiretroviral therapy; IQR, interquartile range.
